# A Biocompatible Synthetic Lung Fluid Based on Human Respiratory Tract Lining Fluid Composition

**DOI:** 10.1007/s11095-017-2169-4

**Published:** 2017-05-30

**Authors:** Abhinav Kumar, Wachirun Terakosolphan, Mireille Hassoun, Kalliopi-Kelli Vandera, Astrid Novicky, Richard Harvey, Paul G. Royall, Elif Melis Bicer, Jonny Eriksson, Katarina Edwards, Dirk Valkenborg, Inge Nelissen, Dave Hassall, Ian S. Mudway, Ben Forbes

**Affiliations:** 1 Institute of Pharmaceutical Science, Faculty of Life Sciences and Medicine, King’s College London, London, SE1 9NH UK; 2 Institute of Pharmacy,, Martin-Luther-Universität Halle-Wittenberg, 06108 Halle (Saale), Germany; 3 MRC-PHE Centre for Environment and Health and NIHR-HPRU in Health Impact of Environmental Hazards, Environmental and Analytical Research Division, Faculty of Life Sciences and Medicine, King’s College London, London, SE1 9NH UK; 4 Department of Chemistry – BMC,, Uppsala University, Uppsala, Sweden; 5 Health Unit, VITO NV, 2400 Mol, Belgium; 6 Interuniversity Institute for Biostatistics and statistical Bioinformatics, Hasselt University, 3500 Hasselt, Belgium; 7 Centre for Proteomics,, University of Antwerp, 2000 Antwerp, Belgium; 8 GSK Medicines Research Centre, Gunnels Wood Road, Stevenage, Hertfordshire, SG1 2NY UK

**Keywords:** aerosol, beclomethasone dipropionate, biopharmaceutics, dissolution, fluticasone propionate, inhalation, solubility

## Abstract

**Purpose:**

To characterise a biorelevant simulated lung fluid (SLF) based on the composition of human respiratory tract lining fluid. SLF was compared to other media which have been utilized as lung fluid simulants in terms of fluid structure, biocompatibility and performance in inhalation biopharmaceutical assays.

**Methods:**

The structure of SLF was investigated using cryo-transmission electron microscopy, photon correlation spectroscopy and Langmuir isotherms. Biocompatibility with A549 alveolar epithelial cells was determined by MTT assay, morphometric observations and transcriptomic analysis. Biopharmaceutical applicability was evaluated by measuring the solubility and dissolution of beclomethasone dipropionate (BDP) and fluticasone propionate (FP), in SLF.

**Results:**

SLF exhibited a colloidal structure, possessing vesicles similar in nature to those found in lung fluid extracts. No adverse effect on A549 cells was apparent after exposure to the SLF for 24 h, although some metabolic changes were identified consistent with the change of culture medium to a more lung-like composition. The solubility and dissolution of BDP and FP in SLF were enhanced compared to Gamble’s solution.

**Conclusion:**

The SLF reported herein constitutes a biorelevant synthetic simulant which is suitable to study biopharmaceutical properties of inhalation medicines such as those being proposed for an inhaled biopharmaceutics classification system.

## Introduction

Drug solubility in lung fluid is an important determinant of the fate of inhaled aerosol medicines. Persistence in the form of a solid particle slows drug availability for target engagement, systemic absorption or metabolism [1–4]. Particles that dissolve slowly provide a sustained release mechanism, but are also susceptible to mucociliary clearance [5] and uptake by macrophages [6], or may lead to drug accumulation [7] or toxicity [8]. These factors, together with recent interest in developing a biopharmaceutical classification system for inhaled medicines [9], have focused attention on drug solubility in the lungs and the need to measure solubility in a medium that is representative of human respiratory tract lining fluid (RTLF). In contrast to intestinal fluid in which drug solubility and dissolution has been investigated extensively [10], the development of lung fluid simulants is in its infancy. Human intestinal fluids have been characterised thoroughly in terms of their composition and structure [11–13]. Simulants have been designed to represent fed and fasted conditions [13,14], studied for their biocompatibility [15] and made available as commercial products [16].

When drug solubility or dissolution in the lungs has been studied, the fluid used to represent RTLF has been water or physiological salt solutions [17] (archetypically Gamble’s solution), often supplemented with phospholipids [18–20] or a surfactant such as sodium dodecyl sulphate (SDS) [21,22]. Alternatively, products based on lung surfactant extracts such as Survanta® or Curosurf® have been used [23]. The tendency to refer to all these fluids as ‘simulated lung fluid’ reflects a confusion regarding how best to simulate RTLF *in vitro*. Emerging data regarding the composition of healthy human lung lining fluid in different regions of the lungs [24] provides an opportunity to design a synthetic lung fluid that incorporates the major or critical components of the fluid that lines the human lungs. For maximum utility, such a simulant should be biocompatible with respiratory cells so that it can be used in models to study lung-particle interactions *in vitro*.

The aim of this study was to characterize a synthetic simulated lung fluid (SLF) that has been developed based on the composition of human RTLF [25]. SLF was manufactured and compared to other fluids which have been used as simulants for RTLF in terms of structure and biocompatibility with A549 alveolar epithelial cells and inhaled drug solubility and dissolution.

## Material and Methods

### Materials

The 25 mg/mL stock solutions of 1,2-dipalmitoyl-sn-glycero-3-phosphocholine (DPPC) and 1,2-dipalmitoyl-sn-glycerol-3-phosphot-rac(1-glycerol) ammonium salt (DPPG) were obtained from Avanti Polar Lipids, Inc. (Alabama, USA). Reagent-grade purified human immunoglobulin (IgG), lyophilized human albumin, Bio-reagent-grade transferrin, Sigma-grade cholesterol, ascorbate, urate, certified reference material-grade glutathione and Hank’s Balanced Salt Solution (HBSS) were supplied by Sigma-Aldrich Company Limited (Dorset, UK). HPLC-grade chloroform and water were supplied by Fischer Chemicals (Loughborough, UK). 6α,9-Difluoro-17-[[(fluoromethyl)sulfanyl]carbonyl]-11β-hydroxy-16α-methyl-3-oxo androsta-1,4-dien-17α-yl propanoate (fluticasone propionate: FP) was purchased from Adooq Bioscience (Irwin, CA), 9-Chloro-11β-hydroxy-16β-methyl-3,20-dioxopregna-1,4-diene-17,21-diyl dipropanoate (beclomethasone dipropionate; BDP) was purchased from Medchem Express (US), and Survanta® from AbbVie Ltd. (UK).

### Preparation of Simulated Lung Fluid (SLF)

The SLF was formulated to contain the most abundant components of healthy human alveolar RTLF in the concentrations that they manifest *in vivo* as determined recently by Bicer [24] and was prepared as described previously [25] (Table I). Briefly, to prepare the liposomal content, 1.92 mL DPPC and 0.2 mL DPPG, from 25 mg/mL stock solutions in chloroform were combined and 5 μL of cholesterol from a 200 mg/mL stock solution in chloroform was added. The mixture was stirred gently in a round bottom flask and the chloroform evaporated under streamed nitrogen gas, to produce a thin layer of lipids at the base of the flask. The proteins were added into the lipid flask in aliquots of aqueous stock solutions: 4 mL of albumin (88 mg/mL), 4 mL of IgG (26 mg/mL) and 1 mL of transferrin (15 mg/mL). In order to represent lung antioxidant levels 88.5 μL of the following antioxidant stock solutions were added: 10 mM ascorbate, 10 mM glutathione, and 5 mM urate in the HPLC-grade water. The mixture was vortexed for 5 min, then gently mixed using a vibrating probe for 10 min at an amplitude of 10 to dissolve the lipids into the solution. Finally, 10 μL of gentamicin was added, followed by 775 μL of HBSS under gentle mixing.

**Table I:** The Composition of Survanta and Simulated Lung Fluid (SLF)

Survanta®	SLF
- Phospholipids 25 mg/mL (including 11.0–15.5 mg/mL disaturated phosphatidylcholine) - Triglycerides 0.5–1.75 mg/mL - Free fatty acids 1.4–3.5 mg/mL - Protein less than 1.0 mg/mL	- DPPC 4.8 mg/mL - DPPG 0.5 mg/mL - Cholesterol 0.1 mg/mL - Albumin 8.8 mg/mL - IgG 2.6 mg/mL - Transferrin 1.5 mg/mL - Ascorbate 140 μM - Urate 95 μM - Glutathione 170 μM

### Characterisation of the SLF

Cryo-transmission electron microscopy (cryo-TEM) of SLF and Survanta was performed using a Zeiss Libra 120 Transmission Electron Microscope (Carl Zeiss NTS, Oberkochen, Germany). The microscope operated at 80 kV in zero loss bright-field mode under cryo conditions. Digital images were recorded under low dose conditions, with a slow-scan CCD camera (TRS GmbH, Moorenweis, Germany) and iTEM software (Olympus Soft Imaging System GmbH, Münster, Germany). An under focus of 1–2 μm was used to enhance the image contrast. Thinly spread samples were prepared in a 100% humidity chamber to avoid dehydration, then quickly vitrified in liquid ethane held at a temperature just above its freezing point (−182°C). After vitrification, the samples were transferred to the microscope using a Gatan CT3500 cryo-transfer (Gatan, Oxon, UK), to maintain samples below −165°C.

Photon correlation spectroscopy (Nanosizer, Malvern Instruments, UK) at a scattered angle of 173° was used to measure the hydrodynamic diameter of structures in SLF and Survanta. Suspensions (1 mL) of both fluids were analysed using instrument parameters: refractive index 1.330, temperature 25°C, dynamic viscosity 0.8882 × 10^−3^ Pa s. Zetasizer Software 6.20 was used to analyze the data.

A Langmuir trough (Model 602A; Nima Technologies Ltd., Coventry, UK) was used to make surface pressure-area measurements at 23°C using a PS4 surface pressure microbalance (0–240 mN/m range, 0.1 mN/m resolution) fitted with a Wilhelmy plate (1 cm width Whatman Grade 1 chromato-graphic paper, GE Healthcare life sciences, Little Chalfont, UK) and controlled by Nima IU4 computer interface unit software. Suspensions of freeze dried SLF and Survanta, 1 mg/mL, were prepared in chloroform, vortexed for 10 min, then bath sonicated at 37 kHz, 25°C, for 10 min. For each isotherm, the test fluid was deposited dropwise onto a 0.9% *w*/*v* NaCl subphase surface using a Hamilton syringe and spreads rapidly to cover the available area of the trough until the surface pressure reached 20 mN/m, with the barriers open at their maximum. The solvent was allowed to evaporate for 10 min before the barriers were compressed at 35 cm^2^/min. The mean molecular weights of the surface-active components of SLF (38,760.9 g/mol) and Survanta (843.5 g/mol) were calculated from their defined compositions and together with the known masses of the deposited monolayers, were used to determine the mean molecular area for each sample. The isotonic saline subphase was used to simulate the influence of normal lung fluid counter-ions on the behavior of the monolayer components [26].

Individual compressions were used to determine the collapse pressure, which was 50 and 60 mN/m for SLF and Survanta, respectively. Subsequently, each film was compressed to a surface pressure 5 mN/m below their collapse pressures and then expanded to reach the initial surface pressure, 20 mN/m. Triplicate experiments of ten isotherm cycles were performed for each simulant without an equilibration period between the expansion and re-compression. Each individual compression-expansion cycle took 10–25 min to complete. The degree of hysteresis was determined from differences between the compression and expansion isotherms for each cycle. The surface compressional modulus (*K*) [27], was calculated using eq. 1:

1 $$ K=\frac{1}{C}=- A\times {\left(\frac{d\varPi}{d A}\right)}_T $$

where *C* is compressibility, A is the mean area per molecule and ${\left(\frac{d\varPi}{d A}\right)}_T$ is the slope of the isotherm at a defined surface pressure. Compressibility is characterized by high surface elasticity and low interfacial stiffness [28]. *K* ranges between 12.5 and 50 mN/m for the liquid expanded phase, 50–100 mN/m liquid intermediate phase and 100–250 mN/m for the liquid condensed phase [29], whilst the condensed state has *K* values >250 mN/m.

### Biocompatibility with the Human A549 Cell Line

Human alveolar epithelial A549 cells (passage 38–46) were cultured in a humidified atmosphere at 37°C, 5% CO_2_ using a cell culture medium (CCM) composed of RPMI-1640 medium supplemented with 10% *v*/v fetal bovine serum (FBS), 1% *v*/v L-glutamine and 0.1% *v*/v gentamicin. For the 3-(4,5-dimethylthiazol-2-yl)-2,5-diphenyltetrazolium bromide (MTT) assay A549 cells were seeded in 96-well plates at 30,000 cells/cm^2^ using reduced serum (2% FBS) CCM for 48 h before exposure to SLF or Survanta for 24 h. After 24 h, cells were washed with PBS and 200 μL of fresh CCM containing 50 μL of MTT solution (2.5 mg/mL in PBS) was added to each well and the plate was incubated for 4 h in a humidified incubator, after which the solution was discarded. The cells were lysed and formazan crystals formed were solubilised with 100 μL of a solution of 10% SDS in dimethylformamide:water (1:1). Cells were incubated with lysis solution overnight at 37°C before the absorbance of solubilised formazan was measured at 570 nm using a SpectraMax microplate reader (Molecular Devices, UK). SLF was non-toxic to A549 cells and was evaluated further using TEM and transcriptomics.

For TEM, A549 cells on glass coverslips were fixed using 2.5% (*v*/v) glutaraldehyde in 0.1 M phosphate buffer for 2 h at 4°C. Following fixation the cells were rinsed with 0.1 M phosphate buffer and treated with 1% (*w*/*v*) osmium tetroxide in 0.1 M phosphate buffer (pH 7.3) for 20 min at 4°C. The coverslips were washed for 10 min in phosphate buffer and dehydrated in a graded ethanol dilution series 0 to 100%. Finally, the cells were infiltrated with TAAB epoxy resin for 4 h at room temperature. To section in the plane of the monolayers, the coverslips were embedded in resin and polymerized for 24 h at 70°C. Ultrathin sections (70–90 nm) were prepared using a Reichert-Jung Ultracut E ultramicrotome, mounted on 150 mesh copper grids, contrasted using uranyl acetate and lead citrate, and examined on an FEI Tecnai 12 transmission microscope operated at 120 kV. Images were acquired with an AMT 16000 M digital camera. Images (*n* ≥ 6 for each sample) were acquired and analysed using ImageJ 1.47 software. Effects on cell health was assessed based on morphometry; cellular, nuclear and mitochondrial area.

For transcriptomic analysis, RNA was isolated from the A549 cells in 6-well plates (30,000 cells/cm^2^ in 2 mL CCM) after 24 h exposure to with SLF or CCM (control). The cells were washed 3 times with PBS, then lysed with RLT buffer (Qiagen). Total RNA was isolated using an RNeasy mini kit (Qiagen) following the manufactures guidelines. The RNA concentration was determined using a NanoDrop spectrophotometer (NanoDrop Technologies, ThermoFisher, USA). RNA integrity was analysed using the Agilent 2100 Bioanalyzer (Agilent Technologies). Only samples demonstrating high RNA Integrity Number value (>9) were qualified for microarray analysis.

RNA was amplified and labeled using the one-colour LowInput QuickAmp Labeling Kit (Agilent Technologies). Briefly, 200 ng of total RNA was reverse transcribed into complementary DNA (cDNA) using a T7-promoter primer and MMLV reverse transcriptase. The cDNA was transcribed into complementary RNA (cRNA), during which it was fluorescently labeled by incorporation of cyanine(Cy)3-CTP. After purification using the RNeasy mini kit (Qiagen), cRNA yield and specific activity were determined using a NanoDrop spectrophotometer. Only labeled cRNAs showing a specific activity above 8 pmol dye/μg RNA were analysed. Labeled cRNA, 1.65 μg, was competitively hybridized onto Whole Human Genome 4 x 44 K oligonucleotide arrays (G4112F, Agilent Technologies) for 17 h in a Tecan HS 4800 Pro Hybridization Station (Tecan Benelux BVBA, Belgium). The arrays were scanned on an Agilent G2565BA microarray scanner and further processed using Agilent Feature Extraction Software (version 10.7.3.1). Data processing steps used to generate the Agilent one-color output was performed as indicated in the Agilent protocol GE1–107-Sep09. For each feature (spot) on the array, gProcessedSignal (normalized values for Cy3 fluorescence), feature quality and gene information were analysed. Normalization included linear scaling and Quantile normalization; data were filtered with regard to reliability including signal strength (only features with signal values 3x the background standard deviation were considered valid features).

### Solubility and Dissolution of Poorly Soluble Inhaled Drugs

The solubility of fluticasone propionate (FP) and beclomethasone dipropionate (BDP) was measured in Gamble’s solution, SLF, Survanta and 0.5% sodium dodecyl sulphate (SDS) by mixing excess drug powder (approximately 0.5 mg) with 0.5 mL of the solvent in a microcentrifuge tube. The sealed tubes were vortex mixed for 5 min before sonication at 37°C for 30 min before transfer to a shaking water-bath at 37°C. After 48 h, the drug suspensions were centrifuged at 13000 rpm for 10 min, then the supernatant (0.2 mL) was centrifuged for a second time before 0.1 mL of supernatant was diluted 10 times with methanol. This sample was analysed for drug concentration by HPLC.

For dissolution experiments Flixotide® 50 μg pMDI or QVAR® 50 μg pMDI were actuated 10 times to deliver aerosol to the surface of 0.45 μm pore polyester membrane Transwell inserts (membrane pre-wetted with dissolution medium) using a twin stage impinge as described previously [30]. The inserts were transferred to wells in 24-well base-plates containing 600 μL dissolution medium (Gambles solution, SLF, or water with 0.5% SDS). At intervals, the insert was moved to a new well containing fresh dissolution medium. The drug transferred to each receiver chamber was measured at the end of the experiment using HPLC as described previously [30]. The test was performed at room temperature, with each result measured in triplicate.

## Results and Discussion

### Characterisation of SLF

**Fig. 1:**
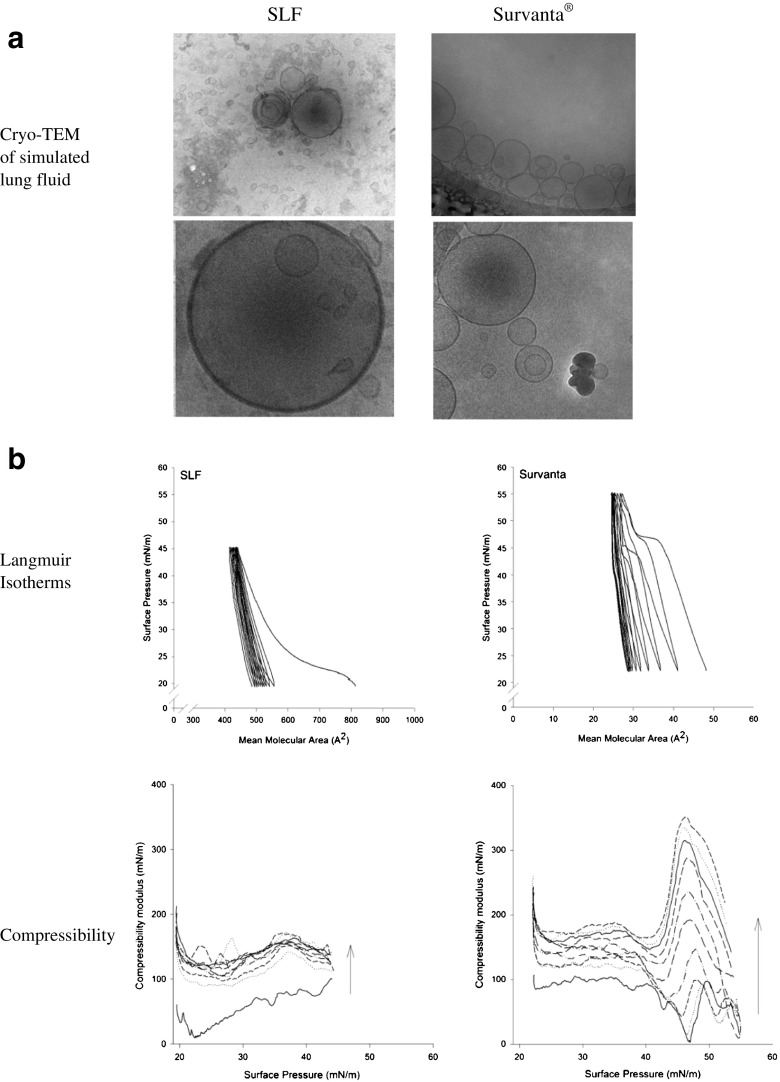
(**a**) CryoTEM. Microstructures in synthetic lung fluid (SLF) and Survanta®. Microstructures imaged in the suspension of SLF and Survanta showing the presence of bilamellar and oligolamellar liposomal vesicles. (**b**) Isotherms. Isotherm cycles and changes in compressibility modulus over 10 consecutive compressions for SLF and Survanta films measured by Langmuir-Blodgett trough.

Uni-, bi-, and oligolamellar liposomes were visualised in the SLF and Survanta using CryoTEM (Fig. 1a and b). The vesicles in SLF and Survanta samples had similar appearance despite their different origins and processing; SLF has a defined compositions and is fabricated from defined individual components, whereas Survanta is an enriched surfactant extract from bovine lungs. Both fluids showed irregular electron-dense structures, which may be protein aggregates. Dynamic light scattering also detected structures in the fluids; SLF showed a strong signal for structures with a size of 57 nm corresponding to the size of liposomes and a weaker signal for 946 nm. Further interpretation was not possible due to background scattering by multi-molecular protein agglomerates, which is a recognized limitation of dynamic light scattering size analysis in media with high protein content [31].

Langmuir isotherms (pressure-area relationships) were explored to determine whether the apparent similarities in structures observed in the lung fluids extended to the physicochemical behavior of monolayers formed on water. The mean molecular area of SLF was greater than that of Survanta (668–800 Å^2^ and 48–26 Å^2^, respectively, over the 10 cycles), possibly due to the albumin (which was present in SLF, but not Survanta) affecting DPPC packing. SLF formed a stable monolayer after the first compression-expansion cycle (Fig. 1b). During compression it underwent transitions from a liquid expanded to intermediate phase, followed by a liquid condensed phase. A large hysteresis loop and shift towards lower molecular area was observed between the first and second compression cycle. The isotherms of the remaining cycles were identical indicating the formation of a stable monolayer.

Three phase transitions were observed in the Survanta isotherm during the first few cycles which disappeared during the later cycles (Fig. 1b). At the initial surface pressure (20 mN/m) Survanta formed an intermediate phase (*K* < 100 mN/m), in which liquid expanded and liquid condensed phases are assumed to co-exist. Upon compression a plateau phase appeared at around 47 mN/m, which occurred at progressively lower surface pressures over the course of subsequent cycles, followed by an intermediate phase when further compression was applied. Over the course of 10 cycles, hysteresis occurred and the isotherm cycles underwent an inward shift. The systematic shift of hysteresis loops implied that there was an irreversible loss of material from the surface due to the irreversible desorption into the subphase over the course of a compression cycle. The reduced hysteresis after the 7th cycle indicates the formation of a stable monolayer at this point from which no further loss of material occurred.

The reduction of Survanta compressibility observed over the sequence of compression cycles has been reported previously and attributed to the extrusion of hydrophobic proteins and associated lipids into the subphase [32]. The hydrophobic SP-B and SP-C proteins present in Survanta are thought to facilitate this extrusion, which is normally reversed upon expansion [33]. However, the speed at which the compression/expansion cycles were repeated may have led to the increasing hysteresis observed in the Survanta isotherms because re-compression began before the monolayer had completed re-spreading. Compared to Survanta, SLF underwent the composition-refining process during the first isotherm cycle when excess material was removed from the interface during the first cycle, indicating a simpler stable colloidal system. These data characterize the structural and mechanical properties of RTLF simulants which may affect drug solubility and dissolution. *In vivo*, a monolayer depleted in hydrophobic proteins during breathing-cycle related compression will show different wetting characteristics for drug particles settling on its surface compared to the expanded system. Thus the first stages of the particle dissolution process will be affected by compression-dependent monolayer composition, whereas changes in the micellar sub phase will influence bulk solubility. *In vitro*, the lower stability of the Survanta monolayer indicates a high propensity to form micellar aggregates which are likely to sequester poorly soluble drugs. In contrast, the stability of colloidal structures in SLF make them less likely to sequester drugs in micelles, however its high albumin content will provide a reservoir for poorly-soluble compounds.

### Biocompatibility of SLF

Survanta had a catastrophic effect on A549 cells in the MTT assay, reducing viability by >90% after 24 h exposure. In contrast, SLF and Gamble’s solution indicated mitochondrial activity comparable to that observed in HBSS with cellular metabolism remaining within 20% of that in reduced-serum CCM control (data not shown). The adverse effects of Survanta were mitigated fully by dilution to 21% Survanta in HBSS (i.e. Survanta diluted to match the phospholipid content of SLF/human RLTF). The apparent biocompatibility of SLF with A549 cells indicated by MTT assay was followed up by exploring any morphometric or transcriptional changes induced by exposure of A549 cells to SLF.

**Fig. 2:**
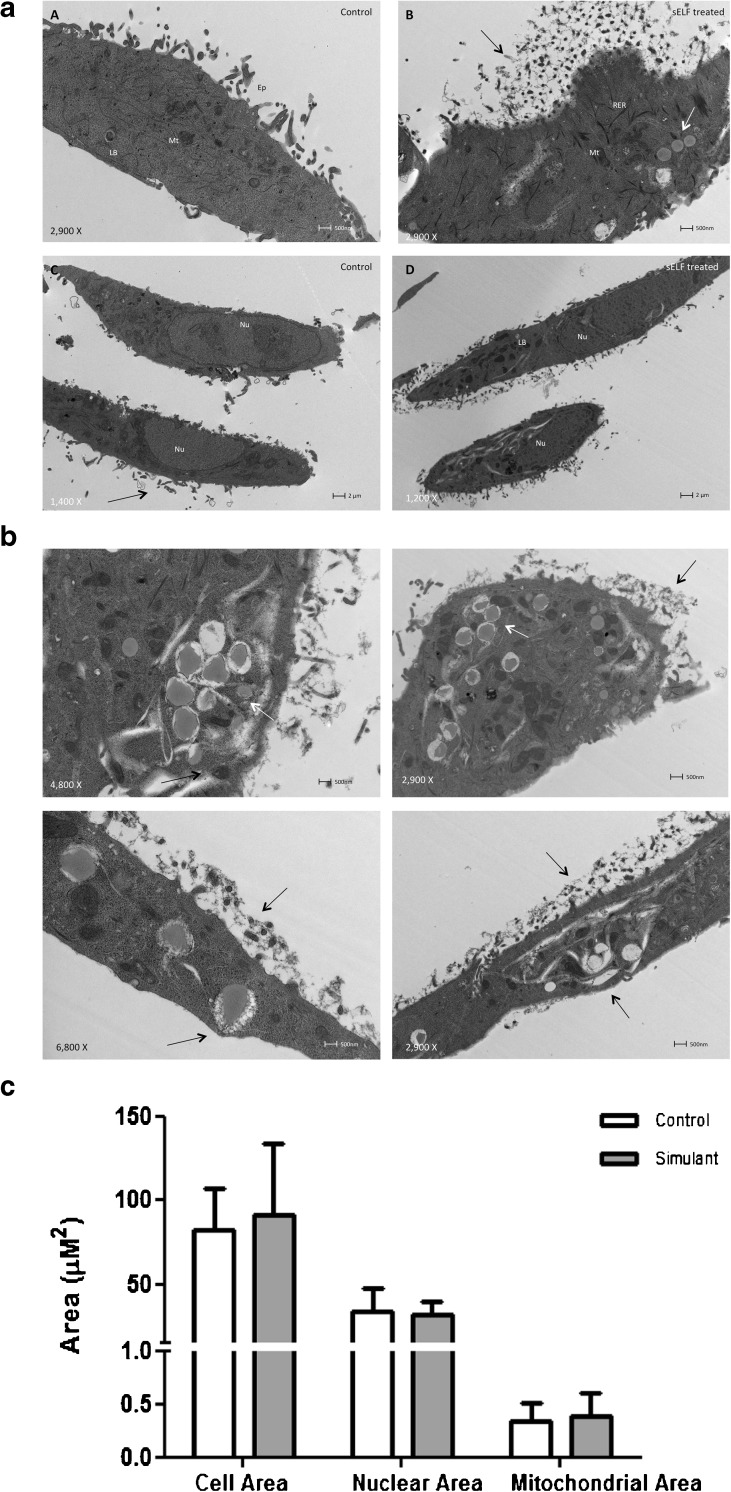
Morphological characterisation of A549 cells cultured in cell culture medium and SLF for 24 h. (**a**) Cell morphology. A549 control cells incubated with cell culture medium (left) compared to A549 cell incubated with SLF (right). Scale bar: 2 μm. (**b**) Cellular uptake. SLF interaction with A549 cells indicated with arrows. Scale bar: 500 nm (**c**) Cell dimensions. Culture medium compared to SLF showing no differences. Data represent mean ± sd, *n* = 6 images.

TEM analysis following incubation of SLF with A549 cells revealed no effect compared to control on cellular, nuclear and mitochondrial areas (Fig. 2). Nor were other morphological signs of cell distress observed, e.g. nuclear condensation, crescent shaped condensed chromatin abnormal lamellar bodies or epithelial projections (Fig. 2a). Interestingly, cytoplasmic and membrane-bound vacuole inclusions (vacuoles at the apical surface of the plasma membrane) were observed, which it is tempting to attribute to uptake of DPPC liposomes following exposure to with SLF (Fig. 2b).

**Fig. 3:**
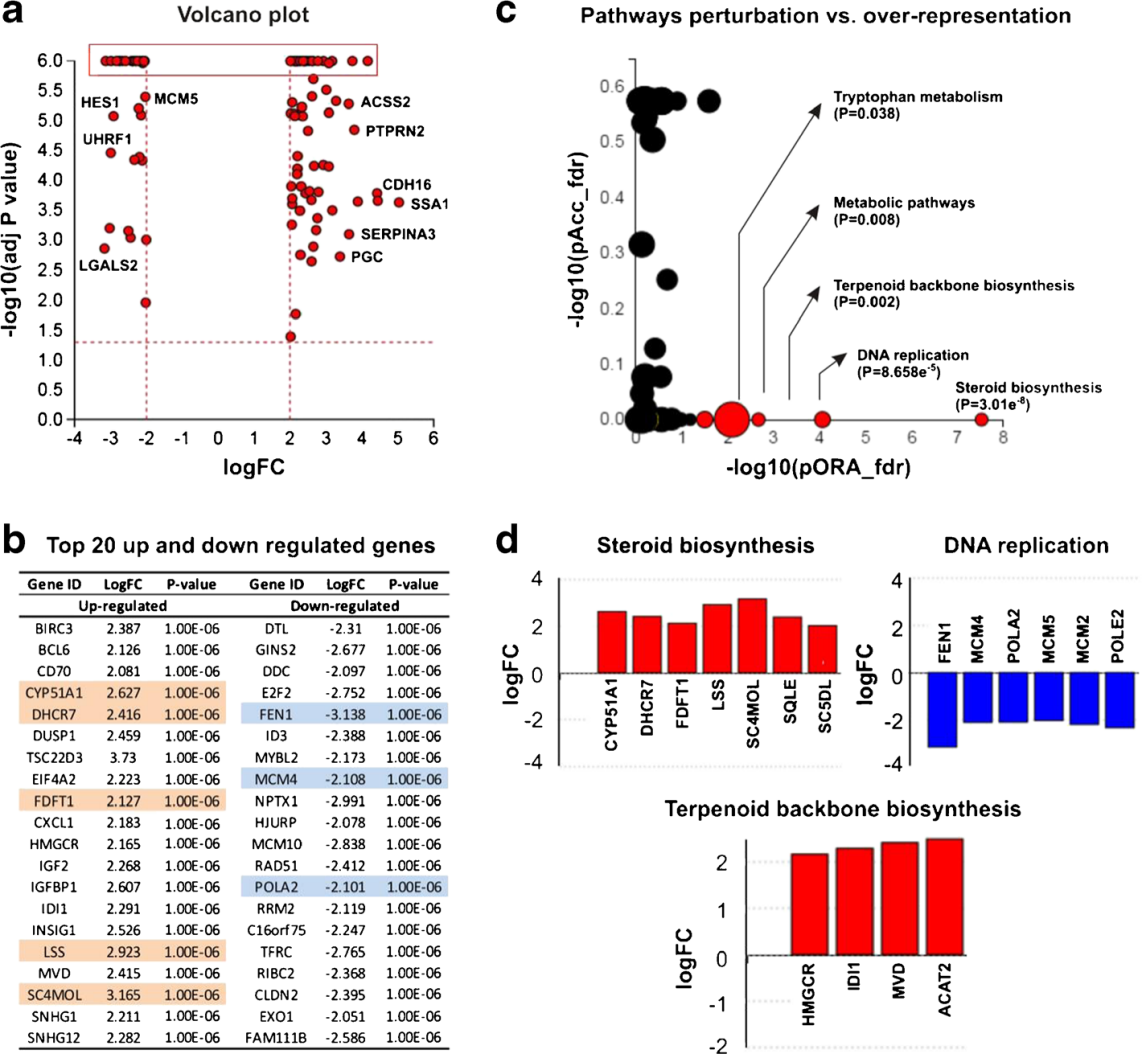
Whole human genome microarray analysis of A549 cells incubated for 24 h with simulated lung fluid (SLF) or standard tissue culture media. Panel (**a**) - Volcano plot: All 116 significantly differentially expressed (DE) genes are displayed according to their measured expression change (x-axis) and negative log (base 10) of the *p*-value (y-axis). The higher the gene is plotted on the y-axis, the more significant it is. The dotted line shows the thresholds for expression change; *p < 0.05*. The top 20 up and down regulated genes, reflecting the highlighted section in panel A are provided in panel (**b**). Panel (**c**) - Pathways perturbation vs over-representation: The most disrupted pathways are plotted in terms of the two types of evidence: over-representation on the x-axis (pORA) and the total perturbation accumulation on the y-axis (pAcc). Red spots indicate significantly perturbed pathways, with the size of the spot reflecting the number of DE genes within the identified pathways. Panel (**d**) illustrates the DE genes within the three most significant pathways identified as perturbed following incubation with the SLF.

Using microarray analysis, 116 differentially expressed genes (*p < 0.05*; 2 log-fold change in expression) were identified out of 10,705 genes with measured expression. The data was analysed using iPathwayGuide (Advaita Corporation, 2015) within the context of pathways in the Kyoto Encyclopedia of Genes and Genomes (KEGG) database and gene ontologies (GO) from the Gene Ontology Consortium database. After correction for the false discovery rate, 5 KEGG pathways were found to be affected significantly (Table II). After elim pruning to favour more significant terms, 5 GO terms with a threshold above 10 genes per term were impacted significantly (Table III). The perturbation (pAcc) provides a perturbation factor for each gene and the over-representation (pORA) describes the probability of changes to the number of genes affected in a certain pathway (Fig. 3).

**Table II:** The Top (Strongest Signal) Pathways in Rank Order with their *p*-values: *p*-values in Bold Represent Significance

Pathway name	Pathway id	*p*-value	*p*-value (FDR)	*p*-value (Bonferroni)
Steroid biosynthesis	00100	**2.599e-10**	**3.015e-8**	**3.015e-8**
DNA replication	03030	**1.493e-6**	**8.658e-5**	**1.732e-4**
Terpenoid backbone biosynthesis	00900	**5.547e-5**	**0.002**	**0.006**
Metabolic pathways	01100	**2.810e-4**	**0.008**	**0.033**
Tryptophan metabolism	00380	**0.002**	**0.038**	0.191
Cell cycle	04110	**0.002**	**0.040**	0.240

**Table III:** Top Gene Outology (GO) Terms and their *p*-values: *p* values in Bold Represent Significance

No pruning				Elim pruning		Weight pruning	
GO Term	*p*-value	*p*-value (FDR)	*p*-value (Bonferroni)	GO Term	*p*-value	GO Term	*p*-value
Sterol biosynthesis process	**2.000e-14**	**5.018e-11**	**5.018e-11**	Cholesterol biosynthesis process	**1.900e-9**	Sterol biosynthesis process	**2.000e-14**
Cholesterol biosynthesis process	**2.300e-13**	**1.924e-10**	**5.771e-10**	Negative regulation of transcription from RNA polymerase II promoter	**0.008**	Cell cycle phase transition	**6.100e-5**
Secondary biosynthesis process	**2.300e-13**	**1.924e-10**	**5.771e-10**	Oxidation-reduction process	**0.013**	Oxidation-reduction process	**0.003**
Steroid biosynthesis process	**1.400e-12**	**8.782e-10**	**3.513e-9**	Sterol biosynthesis process	**0.039**	Negative regulation of transcription from RNA polymerase II promoter	**0.008**
Sterol metabolic process	**1.300e-10**	**6.523e-8**	**3.262e-7**	Steroid biosynthesis process	**0.044**

Pathway analysis showed that steroid biosynthesis, including biosynthesis of cholesterol, steroid hormones and vitamin D, was significantly induced. This was corroborated by the GO analysis which confirmed induction of the cholesterol, sterol and steroid biosynthetic pathways (*p < 0.05*). This may be the consequence of exogenous cholesterol in SLF. An increase in oxidation-reduction processes may also be related to the increase in cholesterol synthesis, which involves many redox enzymes. As many activators of the NfkB pathway are oxidants, the observed up-regulation in the oxidative-reductive process in this study might indicate that incubation with SLF has immunomodulatory roles [34]. This would be consistent with previous findings demonstrating that DPPC can exert anti-inflammatory effects through the inhibition of the NFkB-pathway [35]. Reduced DNA replication was also identified robustly by pathway and GO analysis, i.e. negative regulation of transcription from RNA polymerase II promotor. This may be explained by the effect of change of culture environment which stalls cell replication *in vitro*.

Taken together, the MTT, morphometry transcriptomic analyses demonstrate that SLF is biocompatible with A549 cells, though clearly further work will be required to establish how robust this finding is across cell lines and in primary cell models.

### Solubility and Dissolution of FP and BDP in SLF

**Fig. 4:**
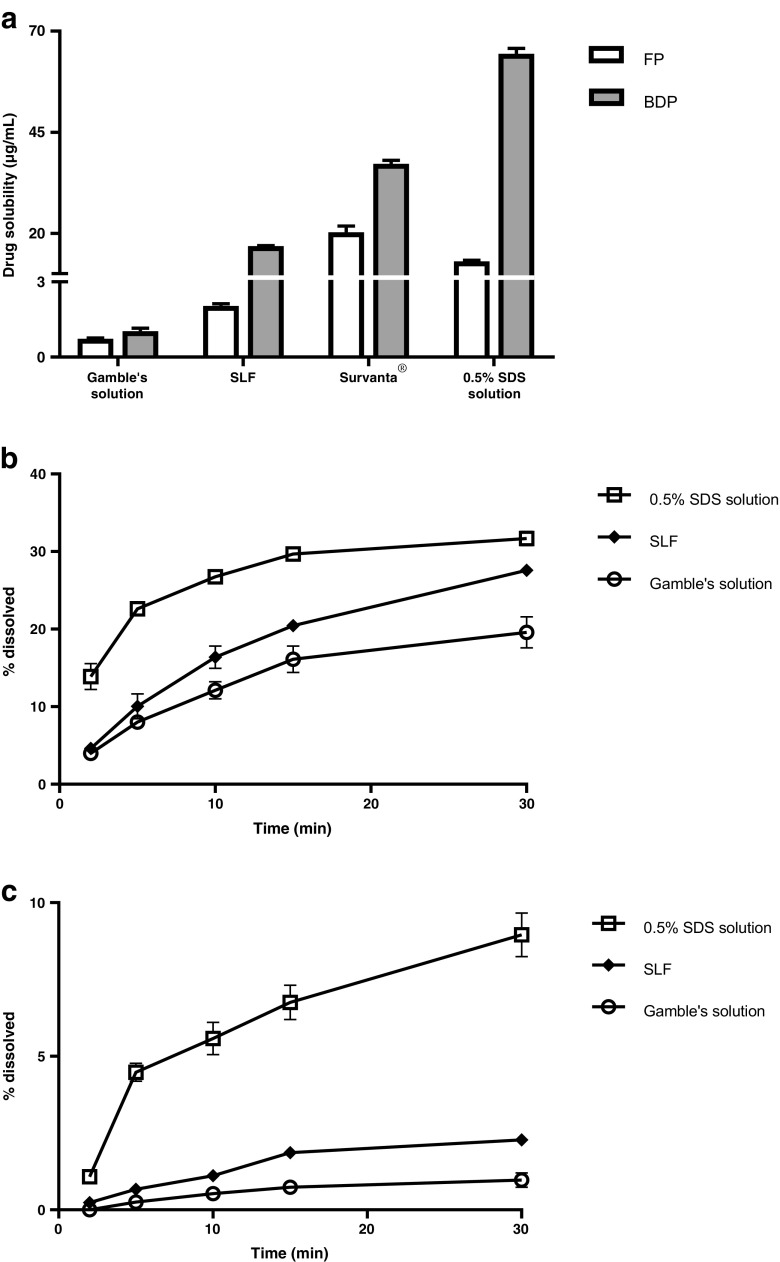
(**a**) Solubility. Beclomethasone dipropionate (BDP) and fluticasone propionate (FP) solubility in media used to represent lung fluid: Gamble’s solution, the biorelevant simulated lung fluid, SLF, Survanta® and 0.5% SDS. (**b**) Dissolution of BDP. Aerosol from QVAR® 50 μg pressurised metered dose inhalers, (**c**) Dissolution of FP. Aerosol from Flixotide® 50 μg pressurised metered dose inhalers. Data represent mean ± sd, *n* = 3.

FP and BDP are poorly water soluble drugs, reported to have concentrations of approximately 0.1–0.2 μg/mL in water [9,36,37]. In Gamble’s solution, FP and BDP solubility was 0.7 and 1.0 μg/mL, respectively (Fig. 4). In surfactant-containing media, the solubility of FP increased in the rank order: SLF (2.0 μg/mL) < 0.5% SDS (13.1 μg/mL) < Survanta (20.3 μg/mL). The BDP rank order of solubility in the different media was changed, but BDP solubility was higher than that of FP in each medium: SLF (16.8 μg/mL), < Survanta (37.2 μg/mL) and <0.5% SDS (64.4 μg/mL). Lung surfactants enhance the solubility of small, lipophilic drug molecules, such as corticosteroids and cationic compounds because they form structures with lipid domains [38,39]. Unexpectedly, FP solubility in SLF was much closer to that in Gamble’s solution than Survanta. Solubilisation may be determined not only by lipid content but also the interaction of components, including the drug, which affect liposomal structures. For example, cholesterol may form tight nanodomain complexes with DPPC stabilising the lamellar structures formed [40], whereas albumin may solubilise the cholesterol [41] and reduce the stability of the lamellar phase and the extent to which drug is solubilised in such structures. A recent review by Das *et al*. speculates how lung surfactant may form different liquid crystalline phases with potential roles in defining dissolution mechanism and rate [42].

The dissolution of FP and BDP aerosols from licenced inhaler products correlated with drug solubility in the dissolution medium. BDP dissolved more readily than FP and for both drugs the dissolution rate in SLF was greater than in Gamble’s solution, but lower than the rate in 0.5% SDS. An estimate for the solubility of FP in the lungs using mechanistic modelling [43] appears to support the value obtained with SLF, supporting the hypothesis that the salt solution is likely to underestimate the dissolution of hydrophobic drugs, while 0.5% SDS may overestimate solubility and hence dissolution in the lungs. FP and BDP were selected to represent inhaled drugs that have poor aqueous solubility and may be dissolution limited, accounting for their relatively slow dissolution profiles which appear to contrast with the rapid onset of action that can be observed for some inhaled molecules, e.g. many bronchodilators. However, it is important to appreciate that oral inhaled product dissolution methods are at an early stage of development and are not optimised for *in vitro-in vivo* correlation. Furthermore, PK-PD relationships to link the potency of inhaled drugs, physicochemical properties, drug formulation, dose interval, temporal profiles of free (unbound) drug concentration at the effect site and pharmacological response are at a nascent stage of development (43, 44).

Using more physiological conditions for *in vitro* investigations into inhalation biopharmaceutics may improve the accuracy of physiologically-based mechanistic modeling and in the future biorelevence may be extended to reflect any differences in RTLF in lung disease and the influence of less abundant components that are recognized to have functional significance, e.g. surfactant proteins that are concentrated in the corona that forms on the surface of biopersistent nanoparticles and influences their uptake [45,46].

## Conclusions

We report a synthetic simulated lung fluid based on human RTLF that can be used for the *in vitro* studies into inhalation biopharmaceutics, e.g. the solubility of inhaled drugs, dissolution of aerosol particles and particle-lung cell interactions. The SLF has a stable colloidal structure, possessing vesicles that are similar in nature to those found in lung extracts. No adverse effects on A549 cells were observed after exposure to the simulant for 24 h, although some metabolic changes were indicated that are consistent with the change of culture medium to a more physiologic composition. Based on preliminary results, we hypothesize that the use of biorelevant medium provides realistic estimates of the solubility and dissolution of hydrophobic drugs *in vivo*. Whilst the present SLF was based on the composition of RTLF from the alveolar region of healthy subjects, we acknowledge that further work is still required to develop simulants reflective of different regions of the airway, as well as variation in composition associated with established respiratory disease.

## Abbreviations

BDP: Beclomethasone dipropionate
CCM: Cell culture medium
DPPC: 1,2-dipalmitoyl-sn-glycero-3-phosphocholine
DPPG: 1,2-dipalmitoyl-sn-glycerol-3-phosphot-rac(1-glycerol)
FDR: False discovery rate
FP: Fluticasone propionate
GO: Gene ontology
HBBS: Hank’s balanced salt solution
KEGG: Kyoto Encyclopedia of Genes and Genomes
MTT: 3-(4,5-dimethylthiazol-2-yl)-2,5-diphenyltetrazolium bromide
pAcc: The total perturbation accumulation
pORA: The over-representation
pMDI: Pressurised metered dose inhaler
RTLF: Respiratory tract lining fluid
SDS: Sodium dodecyl sulfate
SLF: Simulant lung lining fluid
TEM: Transmission electron microscopy
